# Simple Fractures

**Published:** 1899-06-17

**Authors:** 


### SIMPLE FRACTURES.

IN a lecture delivered at St. George's Hospital, Mr. Marmaduke Sheild[1] points out how unfortunate and misleading is the term "simple fracture." The public naturally do not interpret the word in the surgical sense, and if a fracture is said to be simple the patient and his friends regard it as easy to manage and of no grave consequence, whereas in truth a large number of simple fractures are among the most difficult and complicated cases that we have to deal with. In fact, he would go so far as to say that it is nearly time for the surgery of fractures to be rewritten, such a revolution has the introduction of the X-rays worked in their diagnosis, and such aid do they give in their treatment. These rays show us that "simple" fractures are often most serious and complicated injuries, the bones being splintered and cracked in all sorts of irregular ways, so that until the skiagraph is completed it is hardly possible to tell what has happened. In these days, then, one's first duty is to obtain a skiagraph, and if one is in a remote district, and the patient cannot afford the services of an expert with the requisite apparatus, the best plan is to send him to the nearest public hospital. On no account must one guess at the nature of complicated fractures near joints. If it be impossible to obtain the X-rays, the next best thing to do is to examine a complicated case under an anæsthetic, always remembering that a drunken man, with his stomach full of alcohol and food, is a bad subject, and that whatever happens his stomach must be emptied before the anæsthetic is administered. Then, having decided what is the condition, comes the important question whether or not one had better undertake the case without further aid, for it must be remem bered that a poor patient with a very bad fracture is better off in the nearest hospital than in a cottage, and that a doctor may be saved much worry and anxiety, and probably much subsequent ingratitude and reproach, by letting his patient be removed.

The question of consultation again is one that is pretty sure to come up, and it should be faced at once. Mr. Sheild says: "I can only tell you this—with practical experience of the management of fractures, extending now over many years, I never diagnose or treat a bad fracture, especially near a joint, without suggesting a consultation with some hospital surgeon of experience and good repute." The ultimate results of such injuries as a bad "Pott's" fracture, fractures of the upper or lower end of the femur, and fractures into the elbow joint, are often very unsatisfactory. Some deformity, some lameness, some loss of mobility of the articulation persists, and may persist permanently even with the best of skill and care, and a man often cannot be made to understand that subsequent deformity or lameness is due to the severity of the injury and not to lack of skill in treatment; and if he be a foolish or cantankerous person, and there are many such, he naturally wishes to blame someone for his trouble, and the person he will blame will surely be his doctor. His friends and relatives will do likewise, and the aid of the law may have to be invoked for protecting the unhappy practitioner. Now, bearing all this in mind, Mr. Sheild says that it is his practice to write an opinion appropriate to the case and sign it, keeping a copy of the letter by him. In the specimen which he gives of such a communication there occur the following sentences, "If you or your friends should desire some surgeon of repute to see your case, and advise concerning it, now is the time before the bones have joined. . . . I enclose names of surgeons of eminence for you to select from, in case you desire, as I do, consultation upon your case." It is easy to see that the production of a letter of this kind in a court of law might be of the greatest importance in protecting the interests and reputation of the surgeon.

Many of the worst results of simple fracture, such as stiffness and adhesions in the neighbouring joints, implications of bones in inflammatory material, and the adhesion of tendons to their sheaths, are due to the long confinement and the undue interference with the circulation by the pressure of splints and bandages. Accordingly, in cases of fracture where no great displacement occurs, as is notably the case in some fractures of the tibia and femur, daily massage may be employed to the best advantage. But it is best not to commence this before the end of the first week, for the danger of disturbing the newly-formed venous clots, although remote, must not be ignored. In very oblique fractures, however, which start out of position when restraining splints or bandages are removed, it is questionable whether this method can be safely employed until some union has occurred. But, with this reservation, nothing is so important as the methodical and careful use of massage and movement in fractures so soon as ever the bones have united, and the surgeon should see to this himself, and not leave it entirely in the hands of "rubbers." Then when joints remain painful after fractures it may be necessary to treat them by movement under an anæsthetic. Sometimes when this is done an unexpected adhesion gives way, and the functions of the joint are restored as if by magic. It is better that this should be done by the doctor than that some neighbouring bone-setter should get the credit of it.

As a final piece of advice may be mentioned the necessity of taking careful notes of the progress of the case—the date of applying splints, of altering them, of taking them off, the dates of unusual complications, as the onset of delirium tremens, should all be carefully noted, and such notes will be found to be of the greatest help in the event of legal troubles afterwards. One must, in treating fractures, especially since the introduction of the X-rays, always have an eye to possible litigation, and nothing gives a worse impression in a court of law than for a doctor to have to confess that he has taken no notes of his case. When sent for in a hurry to an accident it is no doubt somewhat aggravating to find another medical man before one already in charge of the case, but "you had better retire," says Mr. Sheild, "unless he should ask you to assist him. Rely upon it you do not lose much in the end if a bad fracture falls into other hands." "A badly-united or non-united fracture which you have personally attended is not a pleasant sight to meet constantly limping along the streets of your country town . . . . perhaps for the rest of your life." Clearly those whose ambition runs in the direction of treating fractures had better, as a preliminary, join the Medical Defence Union.

[1] Clin. Jour., May 31.

